# New Appliances and Things Medical

**Published:** 1906-10-06

**Authors:** 


### NEW APPLIANCES AND THINGS MEDICAL.

[We shall be glad to receive at our Office, 28 & 29 Southampton Street, Strand, London, W.C., from the manufacturers, specimens of all new preparations and appliances which may be brought out from time to time.]

### PROTENE ARROWROOT.

(The Protene Company, Limited, 36 Welbeck Street, New Cavendish Street, London, W.)

An important preparation of Protene Arrowroot has been submitted to us. In appearance it resembles a very fine arrowroot, but it is combined with milk proteid to render it more nutritious and more suitable for the delicate digestion of invalids suffering from malnutrition from whatever cause. It is free from irritating matter, and therefore invaluable in the diarrhœa of infants, of catarrh, and in dysenteric affections. When boiled over the fire for two or three minutes, it would form a very useful food for patients with œsophageal stricture. In fact, in any condition where an easily assimilated form of liquid nourishment is required, this preparation can be thoroughly recommended, not only on account of its purity, but for its excellence in manufacture and as a powerful form of nutrition.

### THE PAPKERCHIEF.

(The Papkerchief Syndicate, Limited, 34 Fenchurch Street, London, E.C.)

People ought to be compelled to carry either a piece of rag or a small piece of paper in their pocket to expectorate in. Not only are germs disseminated by the filthy habit, but we have witnessed several elderly ladies nearly thrown down by slipping on expectorated material. The Papkerchief is ideal to carry, as it resembles a pocket handkerchief and is inexpensive and fits the pocket. It is rendered all the more efficient by having a compartment to carry the soiled papers until they can be destroyed by fire. They will keep perfectly dry on account of the waterproof material with which this compartment is lined inside. We have to thank this syndicate for placing on the market such a valuable and inexpensive means of cleanliness and safety both from accident and disease. The Papkerchief holder is an appliance to enable users to carry a supply of papkerchiefs in their pockets and to dispose of the soiled ones in an unoffensive and hygienic way until they can be destroyed, and thus serves a useful and hygienic purpose.

### XEROFORM.

(Chemische Fabrik Von Heyden, Radebeul, Dresden, Germany.)

The preparation before us consists chemically of bismuth-tetraphenol; it is a yellow, tasteless, and odourless powder. It is insoluble in water, and is only decomposed at a temperature of 120° C. Medicinally it is useful as an intestinal antiseptic, its nascent bismuth moiety, as well as its phenol, playing an important part in this action. It is almost non-poisonous, one observer having taken from 75 to 90 grains without any ill-effect. Experiments have been made upon its antiseptic power, and the results have shown that this is greatly superior to iodoform; in fact, some observers say that it has double the bactericidal power of iodoform. It has been largely used as a substitute for iodoform, and, according to the literature before us, with great success. It has also received considerable application in dermatology and dentistry. Internally it may be given to adults in doses from 30 to 75 grains daily. In the case of external application care must be taken to thoroughly cleanse the surface to which it is applied, so that its full efficacy may be developed. It is best used by means of an insufflater or of a wad of absorbent wool. It may be also employed in strengths of from 3 per cent. to 10 per cent. as an ointment or in suspension in oil. The manufacturers also make a gauze containing 10 per cent. of the drug. The trials we have made with this substance confirm the results of the other observers which we have quoted above, and we feel sure that this new antiseptic will find favour with many practitioners.

